# Identification of Normal Blood Pressure in Different Age Group: Erratum

**DOI:** 10.1097/01.md.0000484984.28067.77

**Published:** 2016-06-24

In the article “Identification of Normal Blood Pressure in Different Age Group”,^1^ which appeared in Volume 95, Issue 14 of *Medicine*, Drs. Lin and Wu's affiliations were incorrectly reported originally. The article has since been corrected online.

## References

[1] Lin J-D Chen Y-L Wu C-Z Identification of normal blood pressure in different age group. *Medicine.* 2016 95:e3188. 27057846 10.1097/MD.0000000000003188 PMC4998762

